# Supplementary material for: A Doppler Ultrasound Method to Measure Vascular Responses to Walking
Source: bioRxiv. 2025 Dec 19:2025.12.17.694736. Preprint. [Version 1] doi: 10.64898/2025.12.17.694736 (PMC12724404; doi:10.64898/2025.12.17.694736)
Supplement: 1 — Supplementary Figure S1: Representative plots of raw and filtered A) femoral artery diameter and B) blood flow velocity time-series data from one walking condition. Femoral artery diameter was filtered with a 2nd order recursive Butterworth filter with a 10 Hz cutoff frequency. Blood flow velocity data was filtered using a 5th order 1-dimensional median filter. A 5th order median filter (green line) was best for reducing noise without affecting the signal. Supplementary Figure S2: Peak Leg Blood Flow in the first 20 seconds after each walking condition. Incline walking resulted in the greatest peak Leg Blood Flow compared to Level and Decline walking. [file NIHPP2025.12.17.694736v1-supplement-1.pdf]

bioRxiv preprint doi: https://doi.org/10.64898/2025.12.17.694736; this version posted December 19, 2025. The copyright holder for this preprint (which was not certified by peer review) is the author/funder, who has granted bioRxiv a license to display the preprint in perpetuity. It is made available under aCC-BY 4.0 International license.

40.

41.

42.

SUPPLEMENTAL MATERIAL

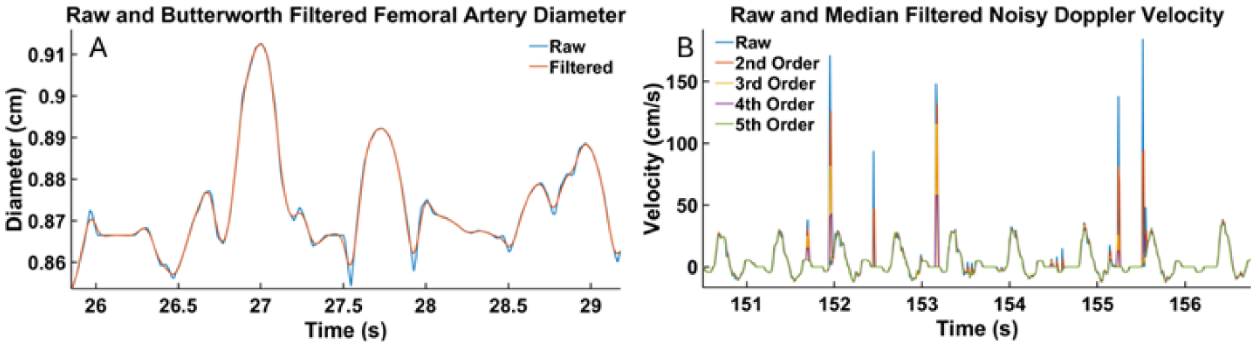

Supplementary Figure S1: Representative plots of raw and filtered A) femoral artery diameter and B) blood flow velocity time-series data from one walking condition. Femoral artery diameter was filtered with a 2nd order recursive Butterworth filter with a 10 Hz cutoff frequency. Blood flow velocity data was filtered using a 5th order 1-dimensional median filter. A 5th order median filter (green line) was best for reducing noise without affecting the signal.

bioRxiv preprint doi: https://doi.org/10.64898/2025.12.17.694736; this version posted December 19, 2025. The copyright holder for this preprint (which was not certified by peer review) is the author/funder, who has granted bioRxiv a license to display the preprint in perpetuity. It is made available under aCC-BY 4.0 International license.

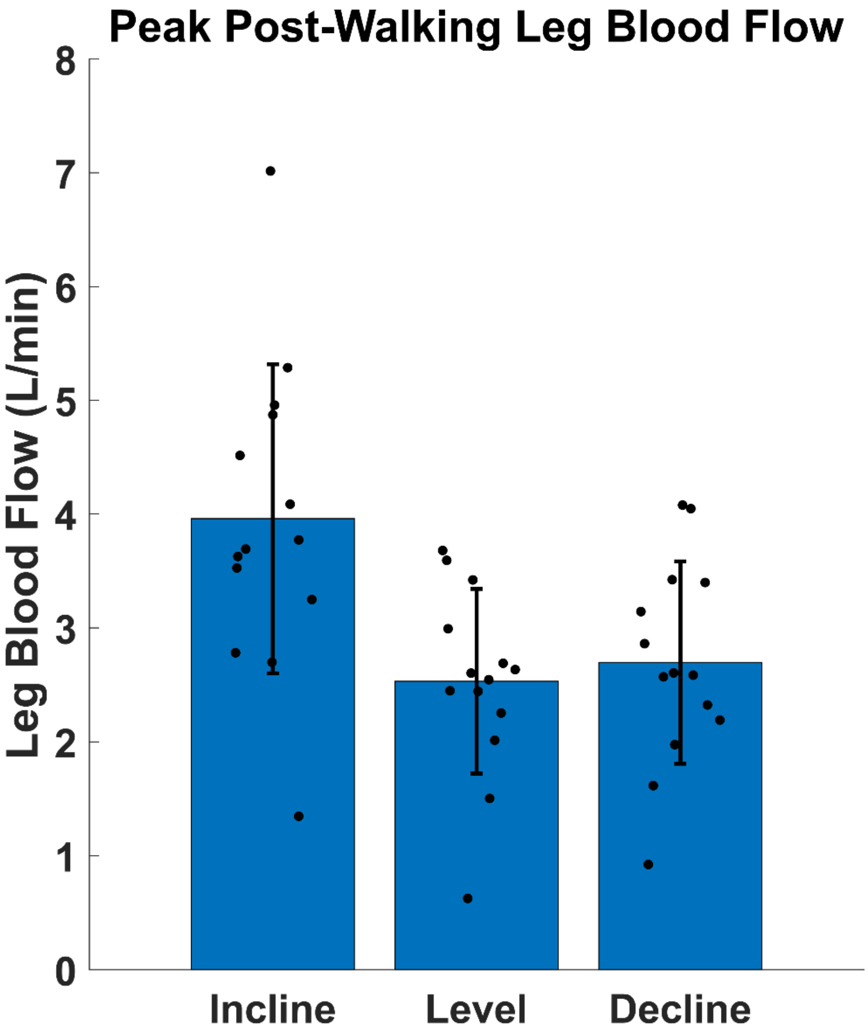

Supplementary Figure S2: Peak Leg Blood Flow in the first 20 seconds after each walking condition. Incline walking resulted in the greatest peak Leg Blood Flow compared to Level and Decline walking.
